# Development of pharmacological strategies for mitochondrial disorders

**DOI:** 10.1111/bph.12456

**Published:** 2014-03-28

**Authors:** M Kanabus, S J Heales, S Rahman

**Affiliations:** 1 Clinical and Molecular Genetics Unit, UCL Institute of Child HealthLondon, UK; 2 Chemical Pathology, Great Ormond Street HospitalLondon, UK; 3 Neurometabolic Unit, National Hospital for NeurologyLondon, UK; 4 Metabolic Unit, Great Ormond Street HospitalLondon, UK

**Keywords:** mitochondrial diseases, models for mitochondrial disorders, treatments for mitochondrial disorders, clinical trials for mitochondrial disorders, nutritional and cofactor support in mitochondrial disorders, mitochondrial biogenesis, gene therapy for mitochondrial diseases

## Abstract

Mitochondrial diseases are an unusually genetically and phenotypically heterogeneous group of disorders, which are extremely challenging to treat. Currently, apart from supportive therapy, there are no effective treatments for the vast majority of mitochondrial diseases. Huge scientific effort, however, is being put into understanding the mechanisms underlying mitochondrial disease pathology and developing potential treatments. To date, a variety of treatments have been evaluated by randomized clinical trials, but unfortunately, none of these has delivered breakthrough results. Increased understanding of mitochondrial pathways and the development of many animal models, some of which are accurate phenocopies of human diseases, are facilitating the discovery and evaluation of novel prospective treatments. Targeting reactive oxygen species has been a treatment of interest for many years; however, only in recent years has it been possible to direct antioxidant delivery specifically into the mitochondria. Increasing mitochondrial biogenesis, whether by pharmacological approaches, dietary manipulation or exercise therapy, is also currently an active area of research. Modulating mitochondrial dynamics and mitophagy and the mitochondrial membrane lipid milieu have also emerged as possible treatment strategies. Recent technological advances in gene therapy, including allotopic and transkingdom gene expression and mitochondrially targeted transcription activator-like nucleases, have led to promising results in cell and animal models of mitochondrial diseases, but most of these techniques are still far from clinical application.

**Linked Articles:**

This article is part of a themed issue on Mitochondrial Pharmacology: Energy, Injury & Beyond. To view the other articles in this issue visit http://dx.doi.org/10.1111/bph.2014.171.issue-8

## Introduction

Recently, the term mitochondrial disorders has become very popular, finding its place in reference to many metabolic diseases, Alzheimer's disease, Parkinson's disease, type 2 diabetes and aging (Patti and Corvera, 2010; Coskun *et al*., 2012; Bratic and Larsson, 2013). In this review, the term mitochondrial diseases denotes a group of inborn errors affecting the oxidative phosphorylation (OXPHOS) system of the mitochondrion. Mitochondrial diseases have a prevalence of approximately 1 in 5000 (Thorburn, 2004). They are a common and unusually heterogeneous group of metabolic disorders, which may present in patients of any age and virtually with any symptoms (Munnich *et al*., 1992). The most important function of OXPHOS is to produce ATP, and therefore, it is not surprising that any defects in this system will be most evident in tissues with high energy demands, for example, the skeletal muscle, brain, liver or heart.

A mitochondrial disorder is part of the differential diagnosis in individuals presenting with myopathy, cardiomyopathy, peripheral neuropathies, stroke-like episodes, seizures, external ophthalmoplegia, sensorineural hearing loss (SNHL), exercise intolerance, diabetes, liver failure, Fanconi syndrome or gastrointestinal symptoms such as nausea, vomiting, pseudo-obstruction, severe dysmotility and faltering growth. These symptoms can occur in isolation or be part of a multisystemic disease presentation (Rahman and Hanna, 2009; Vafai and Mootha, 2012). Currently, there are no effective treatments for the vast majority of mitochondrial disorders and there is an urgent need to develop and evaluate novel therapies. This article discusses the challenges to developing treatment strategies for these complex disorders; models for assessing efficacy of candidate therapies; current therapeutic approaches and the results of previous clinical trials; and the most promising treatments on the horizon, including pharmacological and other strategies for stimulating mitochondrial biogenesis, antioxidant approaches and gene therapy for mitochondrial DNA (mtDNA) and nuclear DNA-encoded mitochondrial disorders.

## Mitochondrial biology and disease

### The mitochondrial respiratory chain (RC) and OXPHOS system

The OXPHOS system is composed of five multimeric complexes (complexes I–IV comprise the RC and complex V is the ATP synthase), which are embedded in the mitochondrial inner membrane. The complexes are composed of multiple subunits, encoded by mtDNA or nuclear DNA. Overall, the RC is composed of approximately 90 proteins. Thirteen of these proteins are encoded by mtDNA, a circular double-stranded DNA molecule, ∼16.6 kb long. Additionally, the mtDNA also encodes 2 rRNAs and 22 tRNAs, which are required for the intramitochondrial synthesis of these 13 proteins. The remaining proteins are translated in the cytosol and transported through the TIM/TOM complex in their precursor form. Once in the correct mitochondrial compartment, the precursor is assembled to form the final protein (Wiedemann *et al*., 2004).

**Figure 1:**
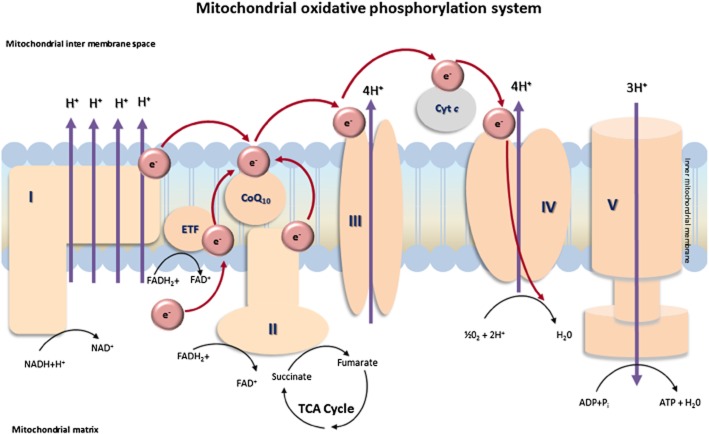
Mitochondrial OXPHOS system. I, NADH : ubiquinone oxidoreductase; II, succinate : ubiquinone oxidoreductase; III, ubiquinol : cytochrome *c* oxidoreductase; IV, COX; V, ATP synthase.

The purpose of the RC is to generate a proton-motive force (complexes I, III and IV are proton-pumping enzymes) across the mitochondrial inner membrane, which is utilized to power ATP synthase, allowing it to produce ATP from ADP and inorganic phosphate – the final step of OXPHOS. Electrons in the form of NADH and FADH_2_, which are derived from the Krebs cycle and β-oxidation, provide the free energy required to pump protons across the membrane. Complex I (NADH : ubiquinone oxidoreductase) oxidizes NADH to NAD^+^ and H^+^ and passes two electrons to ubiquinone (coenzyme Q_10_, CoQ_10_) reducing it to ubiquinol. Simultaneously, complex II (succinate : ubiquinone oxidoreductase), the enzyme that links the Krebs cycle to OXPHOS, oxidizes FADH to FAD^+^ and H^+^ and reduces ubiquinone, while also oxidizing succinate to fumarate, both of which are intermediates of the Krebs cycle. From ubiquinol, electrons are transported to complex III (ubiquinol : cytochrome *c* oxidoreductase), which facilitates the transfer of electrons to cytochrome *c*, which in turn reduces complex IV (cytochrome *c* oxidase, COX). Finally, electrons are donated to molecular oxygen and water is formed (Wallace *et al*., 2010) (Figure 1).

### Mitochondrial genetics and disease

Mitochondrial disorders may be caused by mutations in the mtDNA or in the nuclear DNA. Disorders originating from mtDNA mutations follow a uniparental mode of inheritance, and can only be transmitted from the mother to the child. Disorders caused by mtDNA mutations are complicated by the phenomenon of heteroplasmy (coexistence of mutant and wild-type mtDNA), which arises because of the high copy number of mtDNA. For each mutation, a specific critical threshold of mutation load exists, above which RC function is impaired and disease ensues (Taylor and Turnbull, 2005). Generally speaking, lower mutation load is usually associated with less severe symptoms presenting in adulthood (Rahman and Hanna, 2009).

Mitochondrial disorders due to nuclear DNA mutations follow Mendelian inheritance (DiMauro, 1999). The majority of subunits (∼77), which comprise the mitochondrial RC, are encoded by nuclear DNA. Many additional proteins required for correct functioning of the RC are also encoded by nuclear DNA, which explains why the majority of mitochondrial disorders are caused by mutations in nuclear DNA. Overall, 1500 genes have been suggested as potential causes of mitochondrial disease (Calvo *et al*., 2006), and so far, more than 130 of these have been linked to various mitochondrial disorders (Vafai and Mootha, 2012; Rahman, 2013). These genes are involved in a range of functions including assembly of RC enzyme complexes, maintenance and expression of the mtDNA, cofactor metabolism and biosynthesis and metabolism of mitochondrial membrane lipids. Wide-scale adoption of next generation sequencing approaches has led to a rapid escalation in the number of known disease genes in the last few years, and it is likely that the number of genes linked to mitochondrial disease will soon exceed 200. This huge genetic heterogeneity contributes to the phenotypic heterogeneity of mitochondrial diseases, and presents enormous challenges in devising effective therapies for these disorders.

## Challenges in treatment of mitochondrial diseases

### Difficulties in designing and implementing clinical trials

Development of successful treatments for mitochondrial diseases has proved to be extremely difficult. For other inborn errors of metabolism, therapeutic options include reduction of metabolic load (by dietary manipulation), removal of toxic metabolites, enzyme replacement, enzyme enhancement and organ (liver or bone marrow) transplantation. However, the situation for mitochondrial disorders is rather more complex, for a number of reasons. The main challenges are caused by the extreme genetic and phenotypic heterogeneity of mitochondrial disorders, as discussed above, making it very difficult to collect sufficiently large groups of patients to conduct adequately powered, statistically valid, randomized, double-blinded, placebo-controlled clinical trials. Ideally, clinical trials should be conducted on a group of patients with the same genetic defect, with the same presentation and biochemical findings, at a similar stage of disease progression and, in the case of mutations in mtDNA, a similar mutation load. When dealing with mitochondrial patients collecting a large group of patients that would meet these criteria is virtually impossible in a single centre. Clinical trials that could satisfy these criteria could only exist in the context of multicentre international collaborations (Suomalainen, 2011). A final challenge in implementing clinical trials for mitochondrial disease is a lack of clinically relevant, universally agreed, validated outcome measures.

### Other challenges

Despite enormous progress in understanding mitochondrial biology and mitochondrial diseases in recent years, delivering therapeutic molecules to mitochondria is still challenging. This is mainly due to the relative inaccessibility of the mitochondrion, so that mitochondrially targeted drugs need to pass across several membranes in order to reach the matrix (Heller *et al*., 2012). Some techniques that allow synthetic molecules to be mitochondrially targeted include utilizing the mitochondrial targeting signal peptide, which is a short peptide chain that *in vivo* is attached to proteins that are translated within the cytosol but destined for the mitochondria; conjugating lipophilic cations to small molecules to allow them to accumulate within mitochondria by utilizing the mitochondrial membrane potential; and dequalinium-derived vesicles (so-called DQAsomes) with similar properties to liposomes, which bind DNA and theoretically may be used to deliver small DNA molecules into mitochondria (Yamada *et al*., 2007).

## Recent developments in the therapy of mitochondrial disorders

### Completed clinical trials

Owing to the inherent difficulties in clinical trial design, few randomized double-blinded placebo-controlled trials for mitochondrial disease have been completed. These were the subject of an excellent recent review by Douglas Kerr (Kerr, 2013) and were also critically evaluated in a recently updated Cochrane systematic review (Pfeffer *et al*., 2012). Only 12 studies of 1335 abstracts screened fulfilled Cochrane inclusion criteria for well-conducted unbiased clinical trials (Pfeffer *et al*., 2012; summarized in Table 1). The treatments that have been investigated under such conditions include dichloroacetate (DCA) in five studies and creatine in four studies, either alone or as a cocktail with CoQ_10_ and lipoic acid. Single studies examined the efficacy of CoQ_10_, dimethylglycine and whey-based cysteine supplementation. These clinical trials were mostly conducted in small numbers of patients (9 of the 12 studies included fewer than 17 subjects) and in most cases, the patients had different genetic defects. Dramatic responses were not observed in any of these 12 clinical trials, and no effect was seen in four studies (Table 1). DCA is a pharmacological inhibitor of pyruvate dehydrogenase kinase (PDK), an enzyme that inhibits pyruvate dehydrogenase (PDH). Inhibiting PDK results in reduced lactate levels by maintaining PDH in its active state (Stacpoole *et al*., 1997). Differing results were observed in the five clinical trials of DCA (Table 1) but, importantly, the randomized, controlled clinical trial of DCA in patients with mitochondrial encephalomyopathy (MELAS) had to be terminated because of peripheral nerve toxicity (Kaufmann *et al*., 2006). CoQ_10_ supplementation increased serum CoQ_10_ levels and decreased serum lactate after 1 min of cycle ergometry but other outcome measures were not significantly changed (Glover *et al*., 2010). Combined supplementation with creatine, CoQ_10_ and lipoic acid showed a significant reduction in plasma lactate levels, and slowed the progression of loss of ankle dorsiflexion strength (Rodriguez *et al*., 2007). Dimethylglycine and whey-based cysteine supplementation showed no significant improvement (Liet *et al*., 2003; Mancuso *et al*., 2010). Much has been learned from these completed clinical trials regarding optimization of methodological design and the importance of selecting clinically relevant primary end points. It is encouraging that there was a trend towards larger numbers of participants in the studies conducted in more recent years (Table 1). This is likely to reflect a combination of factors: improved trial design, increased numbers of patients receiving a genetic diagnosis of mitochondrial disease and increased accessibility of clinical trials to patients.

**Table 1:** Randomized, double-blinded, placebo-controlled clinical trials in mitochondrial disease

Treatment	Disease	No. of participants	Type of trial	Outcome	References
CoQ_10_	MELAS, PEO, complex I deficiency, NARP, LHON	30	Randomized, placebo-controlled, double-blind crossover	Serum CoQ_10_ increased, lactate levels decreased after 1 min of cycle ergometry, but no significant change in other endpoints	Glover *et al*., 2010
Creatine	MELAS and MM	7	Randomized, placebo-controlled, double-blind crossover	Increased handgrip strength, NIDT and post-exercise lactate	Tarnopolsky *et al*., 1997
	CPEO and MM	16	Randomized, placebo-controlled, double-blind crossover	No effect	Klopstock *et al*., 2000
	CPEO and KSS	15	Randomised, placebo-controlled crossover	No effect	Kornblum *et al*., 2005
DCA	MM, CPEO, KSS, Leigh syndrome, MELAS	11	Randomised, placebo-controlled, double-blind crossover	Decreased blood lactate, pyruvate and alanine at rest and post-exercise, some improvements in brain MRS	De Stefano *et al*., 1995
	CPEO, MERRF, MM	8	Randomized, placebo-controlled, double-blind crossover	Decreased resting and exercise lactate and pyruvate	Vissing *et al*., 2001
	Mitochondrial RC disorders	9	Randomized, placebo-controlled, double-blind crossover	Decreased blood lactate levels during exercise	Duncan *et al*., 2004
	MELAS m.3243A>G	30	Randomized, placebo-controlled crossover	No effect. Study terminated due to side effects (peripheral neuropathy)	Kaufmann *et al*., 2006
	Congenital lactic acidosis	43	Randomized, double-blinded, placebo-controlled parallel group	Reduced blood lactate levels post high carbohydrate meal	Stacpoole *et al*., 2006
Dimethylglycine	SLSJ-COX	5	Randomized, placebo-controlled crossover	No effect	Liet *et al*., 2003
Whey-based cysteine	PEO	13	Randomized, placebo-controlled, double-blind crossover	Glutathione levels increased. Advanced oxidation protein products and ferric-reducing antioxidant power increased	Mancuso *et al*., 2010
Combination therapy (creatine, α-lipoic acid and CoQ_10)_	CPEO, KSS, MELAS, MNGIE, MM	16	Randomized, placebo-controlled, double-blind crossover	Decreased plasma lactate, slower disease progression (measured by peak angle dorsiflexion strength).	Rodriguez *et al*., 2007

CPEO, chronicprogressive external ophthalmoplegia; MERRF, myoclonic epilepsy, ragged red fibres; NIDT, non-ischaemic, isometric, dorsiflexion torque; SLSJ-COX, Saguenay-Lac-Saint-Jean COX deficiency.

### Outcome measures and natural history studies

When investigating potential therapeutic strategies, the correct choice of outcome measure(s) is very important. For mitochondrial disorders, some of the most commonly measured outcomes include biochemical assessment, such as plasma lactate and pyruvate, and muscle RC enzyme assays; histopathological and histochemical assessment; and neuroimaging including brain MRI and magnetic resonance spectroscopy (MRS) of brain or muscle. However, lactate is not universally elevated in patients with mitochondrial disease and is subject to acute variations so may not be a reliable biomarker in clinical trials. Serial muscle biopsies are invasive; therefore, muscle histology and RC enzyme activities are not practical biomarkers. Neuroimaging also requires general anaesthesia in young children and may not be a sufficiently sensitive method to detect relatively small changes during the period of a clinical trial. Better biomarkers are clearly needed. Fibroblast growth factor-21 (FGF21), a serum cytokine involved in lipid metabolism, has recently been suggested as a potential biomarker for mitochondrial myopathies. In Deletor mice, FGF21 levels correlated with increased COX-negative fibres (Tyynismaa *et al*., 2010), and subsequently, FGF21 was found to be elevated in patients with mitochondrial disorders with skeletal muscle involvement (Suomalainen *et al*., 2011). A composite score can be useful in clinical trials, and wide adoption of the same score would facilitate comparison of clinical trials. Examples of composite scores include the Global Assessment of Treatment Efficacy (GATE) score, which was utilized in two clinical trials of DCA (Kaufmann *et al*., 2006; Stacpoole *et al*., 2006) and the Newcastle paediatric mitochondrial disease scale (NPMDS), which was used in a study of the novel antioxidant EPI-743 (Phoenix *et al*., 2006; Martinelli *et al*., 2012).

Another important recent development is the publication of natural history data from large national cohorts with single gene disorders, such as a nationwide prospective study of nearly 100 Japanese patients with MELAS (Yatsuga *et al*., 2012) and a multinational retrospective study of >50 patients with SURF1 deficiency (Wedatilake *et al*., 2013). These historical data will be vital comparative data for future clinical trials.

### Development of models for mitochondrial disease

Animal and cell models play a crucial role in understanding and developing treatments for many diseases, and especially so for diseases that are as heterogeneous as mitochondrial disorders.

#### Cell models

Initial studies involved cell-based models of mitochondrial disease. Yeast mitochondrial disease models have been used in high throughput screening of drug libraries to identify novel candidate therapies (Schwimmer *et al*., 2006). Patient-derived cell lines, especially lymphocytes and fibroblasts, which can be obtained through relatively non-invasive procedures, are useful models to study mitochondrial function, diseases and potential therapies (Robinson *et al*., 1990). Although there are many benefits in using these cell lines, some considerations need to be taken into account. For example, one of the main drawbacks in using fibroblasts is that some RC deficiencies are tissue specific, and the defect might not be detectable in these cells. Additionally, both fibroblasts and lymphocytes (healthy and diseased) rely on glycolysis as their main source of energy; therefore, RC assays may not show significant differences in enzyme activities in cells obtained from patients compared with controls. In cell culture, substituting galactose for glucose may be used to help combat this issue, forcing cells to rely on OXPHOS as their main source of ATP, thus allowing any enzyme deficiencies to be more apparent (Robinson, 1996). Myoblasts are also a useful model to study mitochondrial diseases since they more closely resemble the affected tissue, but obtaining muscle tissue does require invasive biopsy and (in children) general anaesthesia. An alternative is to use myogenic transdifferentiation of fibroblasts into myoblasts by MyoD transfection (Bulst *et al*., 2012). Generation of induced pluripotent stem cells from patient fibroblasts and reprogramming into neuronal cells will allow the development of more relevant models for neurological mitochondrial diseases (Cherry *et al*., 2013). Further difficulties arise when the cell line is derived from a patient with a mtDNA mutation. As discussed above (section ‘Mitochondrial genetics and disease’) mtDNA mutations are often heteroplasmic with at least two subpopulations being present, one wild type and the other mutated. In patient cell lines, mtDNA populations often drift towards the wild type, although the converse (increasing mutant load) may occur in some instances. Cell lines of higher passage numbers are thus likely to contain less mutant mtDNA (van de Corput *et al*., 1997). Generation of transmitochondrial cybrids with 100% mutant load can overcome this difficulty; these are created by fusing enucleated cells containing mutant mtDNA with a ‘ρ zero’ nuclear donor cell line, which has been depleted of mtDNA (King and Attardi, 1989) and then selecting for cybrids with 100% mutated mtDNA.

#### Animal models

One of the earliest animal models for mitochondrial diseases was a *Drosophila melanogaster* with a technical knockout (TKO) of the gene encoding the mitochondrial ribosomal protein S12. Detailed phenotypic characterization revealed its similarity to mitochondrial sensorineural deafness (Toivonen *et al*., 2001). Other *Drosophila* models have been reported more recently (Fernandez-Ayala *et al*., 2010; Debattisti and Scorrano, 2013). Another animal model for mitochondrial disorders is the nematode *Cenorrhabditis elegans* but only limited phenotypes could be studied in this simple organism, such as biochemical function and effects on survival, motility and reproduction. One study examined riboflavin supplementation in a *C. elegans* model of complex I deficiency (mutation in the *nuo-1* orthologue of the human *NDUFV1* gene), and showed improved assembly and activity of complexes I and IV, increased ATP production, decreased reactive oxygen species (ROS) production and improved general metabolic function (Grad and Lemire, 2006). The last few years have witnessed the development of a plethora of mouse models for mitochondrial disorders (Table 2), generated by various strategies including constitutive and conditional gene KO and mutagenesis with ENU (*N*-ethyl-*N*-nitrosourea). Both mtDNA and nuclear genes have been targeted. Examples of mouse models with mtDNA mutations include the Mito-mouse harbouring a heteroplasmic single mtDNA deletion at high mutation load (Inoue *et al*., 2000), and a mouse model of Leber's hereditary optic neuropathy (LHON) containing the *ND6* P25L human mtDNA mutation (Lin *et al*., 2012). The LHON mouse presents similar features to the human phenotype (Lin *et al*., 2012) making it a useful tool to investigate potential pharmacological and gene therapy treatment approaches for this disease.

**Table 2:** Examples of mouse models for mitochondrial diseases

Gene	Human phenotype	Mouse model	Mouse phenotype	References
*ANT1*	ADPEO (cardiomyopathy in only recessive case reported)	*Ant1 (−/−)*	Ragged-red fibres, mitochondrial proliferation, cardiomyopathy, very high serum lactate levels.	Graham *et al*., 1997
*POLG*	Alpers disease ARPEO ADPEO	*Polg (−/−)* Polg (+/−)	Embryonically lethal. Severe mtDNA depletion. Slight reduction in mtDNA, normal development.	Hance *et al*., 2005
*C10orf2 (PEO1)*	ADPEO MDDS (hepatocerebral)	*Twinkle* ‘Deletor’ mouse	Accumulate multiple mtDNA deletions with progressive COX deficiency and late-onset myopathy.	Tyynismaa *et al*., 2005
*RRM2B*	ADPEO MDDS (encephalomyopathic)	*Rrm2b (−/−)*	Normal at birth, at 6 weeks show growth retardation and die prematurely	Kimura *et al*., 2003
*TK2*	MDDS (myopathic)	*Tk2 (−/−)* *Tk2* (H126N)	Normal at birth, at 7 days show growth retardation, severe hypothermia, severe mtDNA depletion in muscle, heart, liver and spleen. Death at 30 days. Growth retardation, tremor, ataxic gait and severe weakness on day 10. MtDNA depletion.	Akman *et al*., 2008; Zhou *et al*., 2008
*MPV17*	MDDS (hepatocerebral)	*Mpv17 (−/−)*	Adult mice show nephrotic syndrome and chronic renal failure.	Weiher *et al*., 1990
*TFAM*	None reported to date	*Tfam (−/−)*	Embryonically lethal. Severe mtDNA depletion and no detectable OXPHOS.	Larsson *et al*., 1998
*ND6*	Leigh syndrome	*Nd6* (P25L)	Optic atrophy, reduced complex I and increased oxidative stress.	Lin *et al*., 2012
*NDUFS4*	Leigh syndrome	*Ndufs4 (−/−)*	Encephalomyopathy, ataxia at 5 weeks, death ∼7 weeks. Slow growth, lethargy, loss of motor skills, blindness and high serum lactate.	Kruse *et al*., 2008
*NDUFS6*	Fatal infantile lactic acidosis	*Ndufs6 (−/−)*	Cardiomyopathy at 4 months (males) and 8 months (females) and death.	Ke *et al*., 2012
*SDHD*	Paraganglioma	*Sdhd (−/−)* Sdhd (+/−)	Homozygous KO lethal. Heterozygous KO has a decreased Complex II activity.	Piruat *et al*., 2004
*BCS1L*	GRACILE syndrome (cholestasis with iron overload, intrauterine growth restriction, amino aciduria, lactic acidosis and early death), complex III deficiency	*Bcs1l* (S78G)	Failure to thrive, liver steatosis, fibrosis and cirrhosis, tubulopathy, complex III deficiency, premature death.	Leveen *et al*., 2011
*SURF1*	COX-deficient Leigh syndrome	*Surf1 (−/−)*	High rates of embryonic lethality. Reduced birth weight, reduced complex IV activity in muscle.	Agostino *et al*., 2003
*SCO2*	Cardio-encephalomyopathy	*Sco2 (−/−)* *Sco2* (E140K)	Homozygous KO lethal. Complex IV deficiency, no cardiomyopathy and normal life span.	Yang *et al*.,; 2010^2010^
*COX10*	Encephalomyopathy with renal tubulopathy, Leigh syndrome	*Cox10 (−/−)*	Slowly progressing myopathy at 3 months, severe complex IV deficiency.	Diaz *et al*., 2005
*PDSS2*	Encephalomyopathy and nephrotic syndrome, CoQ_10_ deficiency	*Kd/kd (spontaneous mutation)*	Progressive renal failure.	Lyon and Hulse, 1971
*COQ9*	Fatal multisystem disease with CoQ_10_ deficiency	*Coq9 (R239X/R239X)*	Fatal encephalomyopathy.	Garcia-Corzo *et al*., 2013
*HSP40*	None reported to date	*Hsp40 (−/−)*	Dilated cardiomyopathy, RC deficiency and decreased mtDNA levels. Death before 10 weeks.	Hayashi *et al*., 2006

ADPEO, autosomal dominant progressive external ophthalmoplegia; ARPEO, autosomal recessive progressive external ophthalmoplegia.

For nuclear genes, mouse models are now available for RC subunits (e.g. the NDUFS4 and NDUFS6 subunits of complex I, SDHD subunit of complex II and RISP subunit of complex III) and assembly factors (e.g. BCS1L for complex III and COX10, SCO2 and SURF1 for complex IV), and genes involved in maintenance and expression of the mtDNA (Table 2). Mice with defects in mtDNA maintenance genes include KOs of *POLG*, *PEO1* (also known as *C10orf2*, encoding the Twinkle helicase), *TK2* and *RRM2B* (Table 2). Some of these mouse models display embryonic lethality, others relatively little phenotype, but several have clinical phenotypes similar to the respective human diseases, enabling preclinical trials to be performed. The results of trials performed in these mouse models are discussed in the relevant sections below.

### Establishment of national mitochondrial disease consortia

A promising recent advance has been the creation of national consortia aimed at recruiting large cohorts of patients affected by mitochondrial disease. These include the UK MRC Mitochondrial Disease Patient Cohort Study (Nesbitt *et al*., 2013); the Nationwide Italian Collaborative Network of Mitochondrial Diseases (Mancuso *et al*., 2013); and the North American Mitochondrial Disease Consortium (DiMauro, 2013). The existence of these consortia should greatly facilitate recruitment into future clinical trials for mitochondrial disease.

## Current treatment strategies for mitochondrial diseases

### Identification of treatable disorders

One of the most important tasks in the management of mitochondrial disease is the identification of those few disorders that are exquisitely responsive to specific therapies. These include defects of CoQ_10_ biosynthesis, which may present with infantile-onset encephalomyopathy, nephrotic syndrome, SNHL, ataxia, seizures or isolated myopathy (Rahman *et al*., 2012). Early initiation of CoQ_10_ supplementation is related to clinical outcome (Montini *et al*., 2008), but it should be noted that not all patients respond clinically to CoQ_10_ supplementation (Rahman *et al*., 2001; Duncan *et al*., 2009).

Leigh syndrome is almost invariably a devastating progressive neurodegenerative disorder, but rare treatable causes include biotinidase deficiency (which responds to doses of biotin of 5–10 mg·day^−1^), and also the biotin thiamine responsive basal ganglia disease (BTBGD), in which much higher doses of biotin are needed, typically at least 5 mg·kg^−1^·day^−1^ (Alfadhel *et al*., 2013). Patients with BTBGD may also present with an acute Wernicke-like encephalopathy, and need thiamine as well as biotin for optimal clinical response, which is not surprising since the gene mutated in this disorder, *SLC19A3*, encodes a thiamine transporter (Fassone *et al*., 2013).

Recently, we have also observed (secondary) RC enzyme deficiencies in patients with riboflavin transporter disorders in the Brown Vialetto Van Laere spectrum (Foley *et al*., 2013). These patients show clinical improvement following high-dose riboflavin (vitamin B2) supplementation. Riboflavin has also been reported to be beneficial for patients with mutations in *ACAD9*, a gene encoding a flavin-dependent enzyme, which was originally thought to be involved in fat oxidation but which now appears to play a more significant role in complex I assembly (Scholte *et al*., 1995; Gerards *et al*., 2011). Although anecdotal reports of benefit from riboflavin have been reported for patients with other causes of complex I deficiency (Ogle *et al*., 1997), and also some patients with complex II deficiency (Bugiani *et al*., 2006), so far, no clinical trials have been performed to evaluate this formally. Ongoing studies assessing efficacy of riboflavin therapy in two mouse models of nuclear-encoded complex I deficiency may pave the way for clinical trials (Rahman and Thorburn, 2013).

### Supportive therapy

Despite the many and increasingly sophisticated efforts towards finding a suitable cure for mitochondrial disease, currently, most patients are primarily offered symptomatic treatment. Nevertheless, symptom management is important for patients with mitochondrial diseases, improving their quality of life and in some instances prolonging survival (Rahman and Hanna, 2009).

#### General supportive measures

Examples of symptom management approaches include anticonvulsant drugs for mitochondrial seizure disorders (although sodium valproate should be avoided in patients with *POLG* mutations because of the risk of liver failure; Rahman, 2012); eyelid surgery for ptosis; hearing aids and cochlear implants for patients with SNHL; pacemakers and implantable defibrillators for cardiac conduction defects; medical treatment of cardiomyopathies; pancreatic enzymes and insulin for pancreatic failure (particularly seen in patients with Kearns–Sayre syndrome (KSS) and in patients with maternally inherited diabetes and deafness); thyroxine, growth hormone and cortisol replacement in patients with hormonal deficiencies; blood transfusions for patients with Pearson syndrome and other sideroblastic anaemias; electrolyte replacement for patients with significant renal tubular losses (as in many children and young people with KSS); and nutritional support (gastrostomy or even parenteral feeding) for patients with prominent gastrointestinal symptoms including dysphagia, vomiting, diarrhoea and failure to gain or maintain weight (Rahman, 2013).

#### Organ and stem cell transplantation

In situations where the disease targets a single organ such as the heart or liver, organ transplantation can be considered, after careful exclusion of significant neurological involvement (DiMauro and Mancuso, 2007; Rahman, 2013). Allogeneic haematopoietic stem cell transplantation (AHSCT) is currently the only effective way to replace thymidine phosphorylase activity in patients with mitochondrial neurogastrointestinal encephalopathy (MNGIE), and has quickly become accepted as the ‘standard of care’, although it has not yet been evaluated in a clinical trial setting. So far, AHSCT for MNGIE has been associated with ∼50% mortality from disease progression or transplant-related complications (Halter *et al*., 2011). An internationally agreed transplant protocol aims to reduce toxicity of pre-transplant conditioning (Halter *et al*., 2011), and a formal clinical trial is planned (Kerr, 2013).

#### Cerebral folate deficiency

Other supportive therapies are aimed at replacing metabolites that appear to be low in specific subgroups of patients with mitochondrial disease. For example, low CSF levels of the major transport folate 5-methyltetrahydrofolate (5-MTHF) have been documented in several patients with KSS over the last 30 years (Allen *et al*., 1983). The underlying pathogenic mechanisms are debated, but are likely to involve impairment of active transport of 5-MTHF into the CSF, either because of ATP insufficiency or through ROS-mediated damage to the choroid plexus cells responsible for folate transport (Hyland *et al*., 2010; Spector and Johanson, 2010; Serrano *et al*., 2012). Cerebral folate deficiency (CFD) in KSS is associated with cerebral white matter changes, seizures and learning and behavioural difficulties. Treatment with folinic acid led to clinical and radiological improvement in an affected patient (Pineda *et al*., 2006). Further studies are needed to determine which mitochondrial disease patients are at risk of CFD, understand the mechanisms underpinning CFD, and optimize therapeutic protocols for folinic acid replacement and monitoring in these patients.

#### Amino acid supplementation

The amino acids L-arginine, citrulline and taurine have been proposed as potential therapeutic agents in the syndrome of MELAS. Strokes in this condition are thought to result from vascular endothelial dysfunction. The observation of low citrulline levels in some affected patients led to the hypothesis that disturbed nitric oxide homeostasis contributes to the pathogenesis of MELAS (Naini *et al*., 2005) It was therefore suggested that supplementation with arginine, which is required for nitric oxide synthesis, might stabilize vascular function in these patients. A series of open-label studies performed by a Japanese group demonstrated reduced frequency and severity of stroke-like episodes in MELAS patients with the common m.3243A>G mutation (Koga *et al*., 2005; 2006; 2010). More recently, a group at Baylor College has provided preliminary evidence that citrulline may be even more effective than arginine in MELAS (El-Hattab *et al*., 2012). Finally, an open-label study of two Japanese subjects showed amelioration of epilepsy and prevention of strokes following high-dose oral taurine administration over a 9 year period (Rikimaru *et al*., 2012). The authors suggested that providing exogenous taurine reduces the aminoacylation defect associated with the m.3243A>G mutation. Formal double-blinded randomized clinical trials are needed to confirm all of these findings, but are difficult to design and implement because of the episodic and highly unpredictable occurrence of strokes in MELAS.

## Therapies still in development

Despite the disappointing results obtained from the clinical trials performed so far in mitochondrial disease, there are many ongoing studies (*in vitro* and *in vivo*) that provide hope that some pharmacological approaches may be beneficial to subgroups of patients.

### Strategies for increasing mitochondrial biogenesis

**Figure 2:**
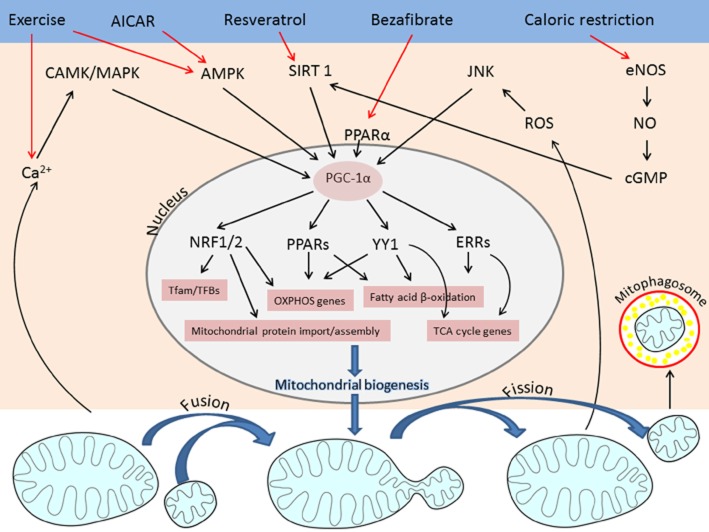
Schematic representation of pathways regulating mitochondrial biogenesis. External factors (exercise, calorie restriction, stress or small molecules such as bezafibrate, resveratrol or AICAR) up-regulate the expression of PGC-1α, which in turn activates NRF1/2, PPAR, YY1 and ERR transcription factors. These are required for up-regulation of key mitochondrial genes including those that encode OXPHOS subunits, Krebs cycle enzymes, fatty acid β oxidation and proteins involved in mitochondrial protein import and assembly.

An approach that has attracted considerable attention is stimulation of mitochondrial biogenesis, with the intention of increasing mitochondrial function simply by having a greater mitochondrial mass. However controversy exists regarding whether increasing biogenesis of damaged as well as normal mitochondria will ultimately be beneficial or harmful. Mitochondrial biogenesis is under complex regulatory control, requiring coordinated transcription of multiple proteins encoded in two cellular compartments. This allows mitochondrial function to be tailored *in vivo* according to nutrient and oxygen availability, hormonal signals, differing metabolic demands and rate of cell proliferation. The transcriptional co-activator PPAR-γ co-activator 1-α (PGC-1α) coordinates mitochondrial biogenesis via a cascade of nuclear-encoded hormone receptors, transcription factors and transcriptional co-activators, including PPARs, oestrogen-related receptors, thyroid hormone receptors, nuclear respiratory factors NRF1 and 2 and the transcription factors CREB and YY1 (see Andreux *et al*., 2013; Dominy and Puigserver, 2013) (Figure 2). In recent years, this signalling cascade has become an attractive therapeutic target to manipulate mitochondrial function, and several methods to increase mitochondrial biogenesis have been explored, including pharmacological strategies, dietary manipulation and exercise therapy.

#### Pharmacological approaches

##### Bezafibrate

PGC-1α overexpression was shown to ameliorate mitochondrial disease in two murine models of COX deficiency: a muscle-specific *Cox10* KO (Wenz *et al*., 2008) and a constitutive *Surf1* KO (Viscomi *et al*., 2011). Following these proof of principle experiments, bezafibrate, a synthetic ligand of PPARα, was used to treat the same mouse models (Wenz *et al*., 2008; Viscomi *et al*., 2011) and also a third mouse model, the Deletor mouse containing a dominant mutation in the Twinkle helicase (Yatsuga and Suomalainen, 2012). The results of bezafibrate treatment differed in these three mouse models. COX activity improved in the muscle-specific *Cox10* KO mice but not in the *Surf1* KO or Deletor mice (Wenz *et al*., 2008; Viscomi *et al*., 2011; Yatsuga and Suomalainen, 2012). Similarly, there was evidence of increased mitochondrial biogenesis in the *Cox10* KO mice but not in the Deletor mice (Wenz *et al*., 2008; Yatsuga and Suomalainen, 2012). A problem with the mouse models is that mice develop abnormal lipid metabolism and hepatomegaly in response to bezafibrate (Viscomi *et al*., 2011; Yatsuga and Suomalainen, 2012). However, this appears to be a rodent-specific effect since bezafibrate has been used safely to treat hyperlipidaemia in hundreds of thousands of people (Prescribing and Primary Care Services and Health and Social Care Information Centre, 2012). Taken together, the results of these studies are promising and suggest that bezafibrate may be beneficial in some mitochondrial disorders. An open-label clinical trial of bezafibrate in the fatty acid oxidation disorder carnitine palmitoyl transferase 2 (CPT2) deficiency was associated with increased physical activity and reduced muscle pain (Bonnefont *et al*., 2010). In view of these encouraging preliminary results, it seems likely that clinical trials in patients with primary mitochondrial diseases will follow in the near future.

##### Resveratrol

Other potential targets to increase mitochondrial biogenesis are the sirtuins, which are NAD+-dependent protein deacetylases whose substrates include PGC-1α and the mitochondrial transcription factor TFAM. Resveratrol activates the sirtuin SIRT1 and has been shown to improve mitochondrial fatty acid oxidation in fibroblasts with defects in CPT2 and very long-chain acylCoA dehydrogenase (Bastin *et al*., 2011). In endothelial cell culture studies resveratrol increased mitochondrial mass and mtDNA content as well the expression of PGC-1α, NRF-1 and TFAM (Csiszar *et al*., 2009). Recently, a phase I trial of a novel SIRT1 activator, SRT2104, appeared to show improved mitochondrial function in healthy elderly human volunteers (Libri *et al*., 2012). However, efficacy of this novel molecule has not yet been investigated in mitochondrial disease states. Ongoing trials include an open-label study of resveratrol in Friedreich's ataxia at the Murdoch Children's Research Institute in Melbourne, Australia (Delatycki, 2012). Friedreich's ataxia is an autosomal recessive degenerative disorder caused in most cases by a GAA triplet expansion in the *FRDA* gene encoding frataxin. Frataxin is involved in regulating mitochondrial iron transport, and *FRDA* mutations result in secondary deficiencies of mitochondrial iron-sulphur cluster containing enzymes (Rötig *et al*., 1997).

##### AICAR

AMP activated PK (AMPK) is a cellular energy sensor (Hardie, 2007) that is another attractive target for modifying mitochondrial bioenergetics in mitochondrial disease. AMPK is activated at high AMP/ATP ratios (i.e. relative energy deficiency states) and acts to phosphorylate several enzymes involved in stimulating catabolism (e.g. by increasing glucose transport and fatty acid oxidation) and inhibiting anabolism (e.g. by reducing glycogen synthesis and lipogenesis). Chronic AMPK activation has also been implicated in transcriptional up-regulation of mitochondrial biogenesis, again via the PGC-1α signalling cascade (Jager *et al*., 2007) since AMPK increases NAD^+^ levels that increase SIRT1 activity (Beher *et al*., 2009; Canto *et al*., 2009), which up-regulates PGC-1α as discussed above. The AMPK activator 5-aminoimidazole-4-carboxamide ribonucleoside (AICAR) increased mitochondrial biogenesis and ATP levels and decreased ROS in human complex I deficient fibroblasts (Golubitzky *et al*., 2011) and has also shown promise in mouse models of COX deficiency (Viscomi *et al*., 2011).

#### Dietary approaches

The ketogenic diet (KD) has also received a lot of attention in recent years as a possible treatment for mitochondrial diseases. The KD is a high-fat, low-carbohydrate diet that appears to have a number of effects. Fatty acid utilization by mitochondrial β-oxidation is stimulated by the KD, leading to formation of ketone bodies, which provide an alternative energy source for the brain, heart and skeletal muscle. Ketone bodies are metabolized to acetyl-CoA, which feeds into the Krebs cycle and thence to the RC and OXPHOS system to ultimately generate ATP, at least partially bypassing complex I. Increased ketone bodies have also been associated with increased expression of OXPHOS genes, possibly via a similar response to starvation. Starvation causes stress to the cell, which results in activation of many transcription factors and cofactors (including SIRT1, AMPK,PGC-1α) that ultimately increase mitochondrial biogenesis (Nunnari and Suomalainen, 2012). The KD has been investigated in both mitochondrial disease cellular and mouse models. Initial studies reported promising preliminary results: KD reduced mutation load of a heteroplasmic mtDNA deletion in a cybrid model (Santra *et al*., 2004), increased the expression levels of uncoupling proteins in mice (Sullivan *et al*., 2004), up-regulated genes involved in mitochondrial biogenesis (Bough *et al*., 2006) and increased mitochondrial glutathione levels (Jarrett *et al*., 2008) in rats. A preclinical trial in the Deletor mouse revealed slowing of mitochondrial myopathy progression in mice treated with the KD (Ahola-Erkkila *et al*., 2010). In humans, there are anecdotal reports of (often transient) benefit of the KD in mitochondrial disease (Malojcic *et al*., 2004; Laugel *et al*., 2007), particularly in patients with epilepsy (Kang *et al*., 2007). The KD has yet to be tested in a randomized, double-blinded clinical trial.

#### Exercise therapy

Exercise therapy was initially explored as a method for ‘heteroplasmy shifting’, that is, to reduce the relative proportion of mutant to wild-type mtDNA in patients with heteroplasmic mtDNA deletions and point mutations (Taivassalo *et al*., 1999). However, open-label clinical studies of aerobic exercise in patients with heteroplasmic mtDNA mutations resulted in a mild increase or no change in mutant mtDNA, yet were associated with clinical benefit in terms of increased exercise tolerance and improved quality of life (Taivassalo *et al*., 2001; 2006). A study of resistance training showed increased muscle strength, again without any significant change in mtDNA mutation load (Murphy *et al*., 2008). A long-term randomized crossover clinical trial is in progress (Haller, 2009). The mechanisms underlying efficacy of exercise in mitochondrial disease are not completely understood. The prevailing hypothesis is that exercise stimulates mitochondrial biogenesis but evidence to support this is currently lacking in mitochondrial disease patients. In one study, there was a baseline increased expression of PGC-1α in patients with mitochondrial myopathies but no further increase was observed following a period of endurance training (Adhihetty *et al*., 2007). This study did demonstrate increased expression of the antioxidant enzyme Mn-superoxide dismutase following training, as well as increases in a mitochondrial import protein and in cytochrome *c*, suggesting that increased antioxidant defence and electron transport may contribute to the adaptive responses following exercise in mitochondrial disease (Adhihetty *et al*., 2007).

It is important to bear in mind that all of the above strategies to increase mitochondrial biogenesis are interrelated as they all converge on PGC-1α and its downstream signalling cascade. Thus, a combination of pharmacological interventions, diet and exercise may be needed to combat mitochondrial disease. However, evaluating such a complex multifactorial intervention in a clinical trial would clearly be extremely challenging.

### Antioxidant approaches

The mitochondrion is the main site of ROS production within the cell. ROS are physiologically important in cell signalling, and in healthy individuals, this is a tightly regulated process. In patients with defects in the RC, however, inefficient transfer of electrons between the four RC complexes results in accumulation of electrons. These can react with O_2_ to form superoxide anions (O_2_^•−^) (Wallace *et al*., 2010). Depending on the precise genetic defect, a defective RC will often lead to increased ROS production, which will further damage the RC complexes. For example, in cultured human astrocytes, complex IV deficiency led to decreased complex II+III activity (Hargreaves *et al*., 2007), which would further exacerbate ROS production. It has been suggested that increased ROS levels are one of the most important factors underlying the development of a phenotype in patients with mitochondrial diseases. The importance of this is highlighted by the observation of severe phenotypes in some patients with relatively mild RC defects, which would not be expected to result in a significant decrease in ATP synthesis. Accordingly, inhibition of the RC increased superoxide formation prior to impairment of cellular energy metabolism in an astrocyte model (Jacobson *et al*., 2005).

As a result of increased ROS levels, GSH levels will also decrease. GSH is the main antioxidant present within mitochondria and functions to metabolize hydrogen peroxide and prevent peroxidation of phospholipids such as cardiolipin (Mari *et al*., 2009). Decreased mitochondrial GSH levels will result in even higher ROS levels and more damage to the RC, resulting in yet more ROS production forming a vicious cycle. Complex IV activity is directly related to the levels of mitochondrial GSH (Heales *et al*., 1996). Increased ROS levels and GSH depletion will result in peroxidation of cardiolipin. Cardolipin is an unsaturated phospholipid, which is important for the formation of supercomplexes as well as the retention of cytochrome *c* within the mitochondrial inner membrane. Supercomplex formation is necessary for the RC enzymes to function effectively (Schagger and Pfeiffer, 2000), while retention of cytochrome *c* within the inner membrane is crucial in the prevention of cellular apoptosis (Orrenius and Zhivotovsky, 2005). In skeletal muscle samples with complex I and/or complex IV deficiency, cytosolic cytochrome *c* levels were found to be higher than in controls (Oppenheim *et al*., 2009). We have demonstrated low GSH in skeletal muscle (Hargreaves *et al*., 2005) and plasma (Salmi *et al*., 2012) samples from patients with RC deficiencies.

Understanding the importance of ROS in the context of mitochondrial disease opens many doors to potential treatments. N-acetylcysteine, a drug currently approved and commonly used in treatment of paracetamol overdose could be beneficial. N-acetylcysteine promotes GSH synthesis by increasing the availability of cysteine, which is rate-limiting for GSH biosynthesis (Ferreira *et al*., 2011). Addition of GSH to skeletal muscle homogenate, even in the presence of oxidizing peroxynitrate, prevented cardiolipin oxidation (Pope *et al*., 2008). A similar finding was observed with the use of Trolox (vitamin E analogue) (Heales *et al*., 1994; Pope *et al*., 2008). Melatonin has also been shown to inhibit cardiolipin oxidization and reduce cytochrome *c* release (Petrosillo *et al*., 2009).

Various antioxidants have been used to treat mitochondrial disease. CoQ_10_ is the most commonly prescribed antioxidant in mitochondrial disease, although a randomized placebo-controlled double-blind crossover clinical trial did not reveal dramatic efficacy of this agent (Glover *et al*., 2010) as discussed above. A phase III clinical trial in children with RC deficiencies is ongoing (Stacpoole *et al*., 2012). Several modifications of the CoQ_10_ molecule have also been investigated. Idebenone has a shorter isoprenyl chain length and is said to have better blood–brain penetrance than CoQ_10_. Several clinical trials have evaluated idebenone in Friedreich's ataxia and there is a suggestion that it may slow neurological progression in this disorder (see Kerr, 2013). A study in LHON did not show a significant change in the primary outcome measure (best eye recovery) but there was a suggestion of improvement in a subgroup (Klopstock *et al*., 2011). A phase IIa (dose-finding) randomized placebo-controlled double-blind trial of idebenone in MELAS is ongoing. MitoQ is CoQ_10_ conjugated to the lipophilic cation triphenylphosphonium, so that it acts as a mitochondria-targeted antioxidant (Kelso *et al*., 2001). MitoQ has been used in mouse models of disorders associated with secondary mitochondrial dysfunction including Parkinson's and Alzheimer's diseases but has not been used in primary mitochondrial diseases.

EPI-743 is a structurally modified variant of CoQ_10_ with bis-methyl groups replacing the bis-methoxy groups on the quinone ring, and a chain length of three isoprenyl units rather than ten. This synthetic molecule was found to be the most potent antioxidant of several hundred structurally modified CoQ_10_ molecules screened in a cell model system, with 1000-fold increased antioxidant properties compared with native CoQ_10_ (Enns *et al*., 2012). EPI-743 was subsequently evaluated in a series of open-label trials in an end-of-life setting in several centres in the United States (Enns *et al*., 2012) and in Rome, and was reported to slow disease progression compared with historical natural history data (Martinelli *et al*., 2012). However, the extremely unpredictable natural history of Leigh syndrome creates great challenges in interpreting data from such open-label studies. A formal randomized double-blind crossover clinical trial of EPI-743 in children with Leigh syndrome is in progress (Klein, 2012).

### Targeting mitochondrial dynamics and mitophagy

The increasing recognition of mitochondria as highly dynamic organelles has led to a new focus for targeting novel therapeutic strategies (Andreux *et al*., 2013). Mitochondrial mass and morphology results from a delicate balance of several interrelated processes, fission/fusion and biogenesis/mitophagy (Figure 2), and modifying each of these processes may have a role in the treatment of different mitochondrial disorders (reviewed by Andreux *et al*., 2013; Stetler *et al*., 2013). Genetically defined disorders of mitochondrial dynamics include defects of mitochondrial fusion caused by mutations in *MFN2* or *OPA1*, presenting as Charcot-Marie-Tooth type 2A and autosomal dominant optic atrophy, respectively (Alexander *et al*., 2000; Delettre *et al*., 2000; Zuchner *et al*., 2004), and impaired mitochondrial fission caused by mutations in *DRP1* and *MFF* (Waterham *et al*., 2007; Shamseldin *et al*., 2012). The recent discovery of specific inhibitors of mitochondrial fusion (M-hydrazone) and fission (MDIVI-1 and P110) may provide therapeutic potential for these disorders (Cassidy-Stone *et al*., 2008; Wang *et al*., 2012; Andreux *et al*., 2013; Qi *et al*., 2013) but preclinical studies are first needed to investigate efficacy and possible adverse effects. These novel agents may also be useful to modify mitophagy in other mitochondrial disorders not primarily caused by fission/fusion defects (Andreux *et al*., 2013).

### Mitochondrial membrane lipids as therapeutic targets

Mitochondrial membrane fluidity and plasticity is intimately related to its lipid content. The first disorder linked to abnormal mitochondrial lipid metabolism was Barth syndrome of dilated cardiomyopathy associated with cyclical neutropenia, growth retardation and 3-methylglutaconic aciduria (Clarke *et al*., 2013). Barth syndrome is caused by abnormal metabolism of cardiolipin (Schlame and Ren, 2006), the most specific phospholipid component of the mitochondrial membrane. Recently four other disorders have been linked to abnormalities of mitochondrial phospholipid biosynthesis or remodelling (Lamari *et al*., 2013): rhabdomyolysis associated with mutations in *LPIN1* (Zeharia *et al*., 2008; Michot *et al*., 2012), Sengers syndrome (hypertrophic cardiomyopathy and cataracts) caused by *AGK* mutations (Mayr *et al*., 2012), MEGDEL (3-methylglutaconic aciduria, SNHL, encephalomyopathy and Leigh-like syndrome) associated with *SERAC1* mutations (Wortmann *et al*., 2012), and congenital muscular dystrophy caused by mutations in *CHKB*, encoding the first step of phosphatidylcholine biosynthesis (Mitsuhashi *et al*., 2011). Targeting mitochondrial lipid metabolism has thus become an attractive therapeutic strategy, not just for these specific disorders, but also for other mitochondrial diseases since altering the mitochondrial membrane lipid composition by dietary manipulation has been shown to affect ATP synthesis, ROS production and the membrane potential (see Monteiro *et al*., 2013).

### Other pharmacological approaches

#### Nucleoside replacement

Mitochondrial DNA depletion syndrome (MDDS) may be caused by nuclear-encoded defects of the mtDNA replication machinery or of nucleoside salvage. Nucleoside imbalance is thought to underlie disease pathogenesis in the latter group, and in theory replacing the deficient deoxyribonucleosides might correct these disorders. Accordingly, addition of dAMP and dGMP prevented mtDNA depletion in deoxyguanosine kinase-deficient patient fibroblasts (Taanman *et al*., 2003) and myotubes (Bulst *et al*., 2009). A potential concern regarding the use of this form of therapy *in vivo* is that nucleoside pools are tightly regulated and theoretically replacing one or two nucleosides may result in iatrogenically induced nucleoside imbalance and thereby exacerbate the mtDNA replication defect in affected patients. Recently, it has been proposed that simultaneous administration of deoxyribonucleosides and inhibitors of their catabolism may avoid unwarranted side effects (Camara *et al*., 2013). Further studies are in progress.

#### α-Lipoic acid

α-Lipoic acid is a cofactor of three mitochondrial enzymes (PDH, α-ketoglutarate dehydrogenase and branched chain ketoacid decarboxylase) and was first tried as a treatment for PDH deficiency nearly 25 years ago (Byrd *et al*., 1989). Recently, it has been shown that a subgroup of patients with abnormal mitochondrial energy metabolism have defects in lipoic acid synthesis (Mayr *et al*., 2011). It is possible that treatment with lipoic acid may benefit this group of patients, although no formal studies have been reported yet. As discussed above, a trial of lipoic acid in combination with creatine and CoQ_10_ in a heterogeneous group of mitochondrial disease patients showed only mild benefit (Table 2; Rodriguez *et al*., 2007).

#### Enzyme replacement therapy

Unlike lysosomal storage disorders, for which recombinant enzyme replacement therapy (ERT) has been approved or is in clinical trial for more than 10 different disorders (Desnick and Schuchman, 2012), no ERTs are currently available for mitochondrial disorders. However, enzyme replacement has been attempted for MNGIE using purified thymidine phosphorylase enzyme encapsulated in the patient's own red blood cells (Moran *et al*., 2008), so far without long-term clinical success. Very recently, a preclinical toxicity evaluation of this system has been performed in mice and Beagle dogs, and was complicated by infusion-related immune responses in both species (Levene *et al*., 2013). Finally, one study described the construction of active thymidine phosphorylase encapsulating nanoreactors as novel enzyme delivery vehicles (De Vocht *et al*., 2010) but clinical utility of this method has not yet been reported in either cell or animal models of MNGIE.

#### Miscellaneous approaches

Other specific therapies that have been proposed include bypass of complex I using succinate, which is metabolized via complex II (Oguro *et al*., 2004) or triacylglycerol supplementation (metabolized via fatty acid oxidation, resulting in FADH_2_ production, which feeds into complex II). Triacylglycerol infusion in four patients with complex I deficiency resulted in increased exercise tolerance (Roef *et al*., 2002b) but no effect on plasma lactate levels (Roef *et al*., 2002a). Metronidazole has been used to reduce sulphide production by intestinal anaerobes in ethylmalonic encephalopathy, a disorder of sulphur detoxification (Viscomi *et al*., 2010). N-acetylcysteine has also been used in the same disorder, in order to replenish GSH as a means of buffering sulphide (Viscomi *et al*., 2010).

### Gene therapy

Although this review is primarily focussed on pharmacological strategies, it is important to mention some recent potentially exciting developments in gene therapy approaches for mitochondrial diseases. Gene therapy for mtDNA defects is particularly challenging since therapeutic DNA molecules need to pass across the two mitochondrial membranes in addition to the plasma membrane. Furthermore, mtDNA gene therapy strategies also need to take into account the high copy number of mtDNA, with thousands of copies per cell.

#### Allotopic gene expression

Recoding genes that are normally encoded by mtDNA so that they can be inserted into and expressed from the nucleus is known as allotopic gene expression. This technique was used successfully to transfer the recoded mitochondrial *MTATP6* gene and thereby rescue the ATP synthesis defect in cybrids containing the m.8993T>G mutation, which is associated with maternally inherited Leigh syndrome and neuropathy, ataxia and retinitis pigmentosa (NARP) (Manfredi *et al*., 2002). Furthermore, allotopic expression of the *MTND*4 gene prevented blindness in a rat model of LHON (Manfredi *et al*., 2002; Ellouze *et al*., 2008). A human clinical trial of allotopic gene therapy in LHON is in progress but has not yet been reported (Lam *et al*., 2010).

#### Transkingdom gene therapy

Several studies have used transkingdom gene therapy, where a gene from one species is used to correct a disorder in another species, to target mitochondrial diseases. Transkingdom gene therapy was first suggested as a possible treatment for complex I deficiencies in 2006 (Yagi *et al*., 2006) and subsequently adeno-associated virus (AAV) tagging of the yeast alternative NADH dehydrogenase NDI1 was used to treat an animal model of LHON (Marella *et al*., 2010). Similarly, the yeast alternative oxidase bypasses complexes III and IV and was shown to rescue COX deficiency in human cultured cells (Dassa *et al*., 2009) and a mouse model (El-Khoury *et al*., 2013). Transgenic expression of *D. melanogaster* deoxyribonucleoside kinase (Dm-dNK) was able to rescue myopathic MDDS in mice with thymidine kinase deficiency (Krishnan *et al*., 2013).

#### Other gene therapy approaches for mtDNA defects

DNA delivery into the mitochondrion has been attempted using liposome-based nanocarriers such as Mito-Porter (Yasuzaki *et al*., 2010) or a DQAsome transfection system (Lyrawati *et al*., 2011), delivery of cytosolic tRNAs into mitochondria as a method to aid mitochondrial translation (Mahata *et al*., 2006) and using restriction enzymes to specifically degrade mutant mtDNA (Tanaka *et al*., 2002). Heteroplasmy shifting has also been achieved using antisense oligonucleotides in cybrids containing a heteroplasmic mtDNA deletion (Comte *et al*., 2013). Very recently, a new approach used transcription activator-like effector nucleases engineered to localize to mitochondria, to eliminate mutant mtDNA from cybrids containing the m.14459G>A mutation associated with LHON plus dystonia (Bacman *et al*., 2013). The efficacy of these approaches is strongly debated, and preclinical studies will be required to ensure the safety of these novel agents in whole organisms.

#### Gene therapy for nuclear defects

Gene therapy for nuclear-encoded mitochondrial disorders is technically less challenging and AAV and lentiviral-mediated gene therapy has been performed in murine models of ethylmalonic encephalopathy and MNGIE, respectively, with encouraging results (Torres-Torronteras *et al*., 2011; Di Meo *et al*., 2012). Similar approaches would be applicable for other nuclear-encoded mitochondrial diseases.

#### Pronuclear and spindle cell transfer

Germ line gene therapy has been proposed for mtDNA mutations using the techniques of pronuclear transfer and maternal spindle cell transfer, as a potential method for preventing transmission of mutated mtDNA from the mother to the embryo. In the case of pronuclear transfer, donor and recipient zygotes are enucleated and the recipient's nucleus is then fused into the enucleated donor zygote. This has been demonstrated to be technically possible in research using abnormally fertilized human embryos (Craven *et al*., 2010). Maternal spindle cell transfer (MSCT) involves transfer of nuclear material between donor and recipient unfertilized metaphase II oocytes, and several Rhesus monkeys have been born from oocytes manipulated in this way (Tachibana *et al*., 2009). More recently, MSCT has been shown to be an effective method to replace mtDNA in human oocytes (Tachibana *et al*., 2013). There has been considerable discussion regarding the ethics surrounding these procedures (Bredenoord and Braude, 2010; Nuffield Council on Bioethics UK, 2012), but recently, after an extensive public consultation process (Pitts-Tucker, 2012), the UK government has granted permission for further human research to be conducted using these techniques (House of Commons Hansard Debates 25 June, 2013).

Although all of these studies give promise for future therapies, they are still very far from being offered to patients.

## Conclusions

The complexity of the mitochondrial organelle and the disorders associated with its dysfunction creates unusual challenges for developing effective treatments. There are particular difficulties in clinical trial design for this group of heterogeneous disorders with unpredictable clinical courses. The few well-designed clinical trials that have been conducted to date have failed to identify any clearly effective treatments for mitochondrial disease. However, recent developments including the establishment of national and international consortia aimed at collecting large well-characterized cohorts of patients affected by mitochondrial disease, the availability of mouse models of numerous mitochondrial disorders, the identification of novel drug targets including components of the complex signalling cascades controlling mitochondrial biogenesis, and the realization of novel gene therapy approaches, all herald promise for developing and testing new mitochondrial therapeutic agents. Given the extreme clinical, biochemical and genetic heterogeneity of mitochondrial disease, it is extremely unlikely that a ‘one size fits all’ universal panacea exists. Rather, it is more likely that different interventions will be effective for different subgroups of mitochondrial diseases. Ultimately, it is hoped that it will be possible to deliver a ‘personalized medicine’ approach to patients affected by mitochondrial disease, with the goal of long-term survival with good quality of life.

## Abbreviations

AAV: adeno-associated virus
AICAR: 5-aminoimidazole-4-carboxamide ribonucleotide
CFD: cerebral folate deficiency
COX: cytochrome c oxidase
CPT2: carnitine palmitoyl transferase 2
DCA: dichloroacetate
ERT: enzyme replacement therapy
FGF21: fibroblast growth factor-21
KD: ketogenic diet
KO: knockout
KSS: Kearns–Sayre syndrome
LHON: Leber's hereditary optic neuropathy
MDDS: mitochondrial DNA depletion syndrome
MELAS: mitochondrial encephalomyopathy, lactic acidosis, and stroke-like episodes
MNGIE: mitochondrial neurogastrointestinal encephalopathy
MRS: magnetic resonance spectroscopy
MSCT: maternal spindle cell transfer
mtDNA: mitochondrial DNA
NARP: neuropathy, ataxia and retinitis pigmentosa
OXPHOS: oxidative phosphorylation
PDH: pyruvate dehydrogenase
PDK: pyruvate dehydrogenase kinase
PGC-1α: PPAR γ co-activator 1-α
RC: respiratory chain
ROS: reactive oxygen species
